# Inulin-Type Fructans Modulates Pancreatic–Gut Innate Immune Responses and Gut Barrier Integrity during Experimental Acute Pancreatitis in a Chain Length-Dependent Manner

**DOI:** 10.3389/fimmu.2017.01209

**Published:** 2017-09-26

**Authors:** Yue He, Chengfei Wu, Jiahong Li, Hongli Li, Zhenghua Sun, Hao Zhang, Paul de Vos, Li-Long Pan, Jia Sun

**Affiliations:** ^1^State Key Laboratory of Food Science and Technology, Jiangnan University, Wuxi, China; ^2^School of Food Science and Technology, Jiangnan University, Wuxi, China; ^3^Division of Medical Biology, Department of Pathology and Medical Biology, University of Groningen, University Medical Center Groningen, Groningen, Netherlands; ^4^School of Medicine, Jiangnan University, Wuxi, China

**Keywords:** dietary fibers, inflammation, pancreatic–intestinal immunity, signaling kinases, tight junction proteins, antimicrobial peptides

## Abstract

Acute pancreatitis (AP) is a common abdominal inflammatory disorder and one of the leading causes of hospital admission for gastrointestinal disorders. No specific pharmacological or nutritional therapy is available but highly needed. Inulin-type fructans (ITFs) are capable of modifying gut immune and barrier homeostasis in a chemistry-dependent manner and hence potentially applicable for managing AP, but their efficacy in AP has not been demonstrated yet. The current study aimed to examine and compare modulatory effects of ITFs with different degrees of fermentability on pancreatic–gut immunity and barrier function during experimentally induced AP in mice. BALB/c mice were fed short (I)- or long (IV)-chain ITFs supplemented diets for up to 3 days before AP induction by caerulein. Attenuating effects on AP development were stronger with ITF IV than with ITF I. We found that long-chain ITF IV attenuated the severity of AP, as evidenced by reduced serum amylase levels, lipase levels, pancreatic myeloperoxidase activity, pancreatic edema, and histological examination demonstrating reduced pancreatic damage. Short-chain ITF I demonstrated only partial protective effects. Both ITF IV and ITF I modulated AP-associated systemic cytokine levels. ITF IV but not ITF I restored AP-associated intestinal barrier dysfunction by upregulating colonic tight junction modulatory proteins, antimicrobial peptides, and improved general colonic histology. Additionally, differential modulatory effects of ITF IV and ITF I were observed on pancreatic and gut immunity: ITF IV supplementation prevented innate immune cell infiltration in the pancreas and colon and tissue cytokine production. Similar effects were only observed in the gut with ITF I and not in the pancreas. Lastly, ITF IV but not ITF I downregulated AP-triggered upregulation of IL-1 receptor-associated kinase 4 (IRAK-4) and phosphor-c-Jun N-terminal kinase (p-JNK), and a net decrease of phosphor-nuclear factor kappa-light-chain-enhancer of activated B cells (NF-κB) p65 (p-NF-κB p65) nuclear translocation and activation in the pancreas. Our findings demonstrate a clear chain length-dependent effect of inulin on AP. The attenuating effects are caused by modulating effects of long-chain inulin on the pancreatic–gut immunity via the pancreatic IRAK-4/p-JNK/p-NF-κBp65 signaling pathway and on prevention of disruption of the gut barrier.

## Introduction

Acute pancreatitis (AP) is a sudden inflammation of the pancreas caused by inappropriate activation of local digestive enzymes. During the past decades, it has become the leading cause of hospital admission for gastrointestinal disorders in many countries and the worldwide incidence is increasing (1). Although the mild form of AP is self-limited, nearly 20–25% of patients with AP develop severe symptoms with systemic inflammatory responses. The mortality rate in these patients is as high as 30% (2). Up to now, a targeted pharmacological or nutraceutical therapy specific for AP management is lacking (3, 4). Novel insight however in function–effector relationship of dietary components and their anti-inflammatory effects has generated optimism about the possibility to create nutraceutical strategies to control local inflammation and prevent systemic complications of AP (2).

Pathophysiological studies of AP have revealed that AP is caused by unregulated intra-acinar activation of trypsin and other digestive enzymes, leading to autodigestion of the pancreas and local inflammation. The main triggering factors include pancreatic hyperstimulation, gallstones, and alcohol abuse (5). The induced local inflammation is accompanied by activation of endothelial cells and transendothelial migration of leukocytes, neutrophils, and macrophages, leading to release of harmful enzymes and cytokines that amplify the local inflammatory responses. As a consequence, pancreatic complications such as acinar cell necrosis, pseudocyst formation, and abscess development might occur in most severe cases, leading to transmission of inflammation to other remote organs, which is ultimately responsible for AP-associated mortality.

Innate immune activation and acinar cell inflammatory signaling play a pivotal role in the pathogenesis of AP (5–8). Local inflammation is initiated by acinar cell damage and local production of pro-inflammatory cytokines/chemokines by these cells, followed by infiltration of neutrophils and macrophages (9). Neutrophils, macrophages, and dendritic cells with distinct cell-surface and intracellular markers have been associated with the development of AP and severity of the inflammatory conditions (10–13). The balance between pro-inflammatory and anti-inflammatory mediators produced from acini and infiltrated immune cells determines the outcome of the AP. Tumor necrosis factor (TNF)-α, interleukin (IL)-1, and IL-10 are important cytokines in AP (11, 14–16). TNF-α is released by local Ly6C^hi^ monocytes/macrophages in the pancreas and enhances the severity of the experimental disease (11, 17, 18). Regulatory cytokines, such as IL-10, limit the local and systemic consequences of experimental pancreatitis (18). Expression of these cytokines is under the control of the transcription factor NF-κB. NF-κB activation in pancreatic acinar cells is responsible for the expression of a large number of inflammation-related genes (19). NF-κB activation is regulated by its upstream signaling kinases, such as MAP kinases (MAPKs), which may be activated by receptor-associated kinases, such as IL-1 receptor-associated kinase 4 (IRAK-4) and transforming growth factor-β activated kinase (TAK) (20, 21). Consequently, modulation of immune cell activation, inflammatory cytokine production, and pancreatic inflammatory signaling molecules may be rational approaches to alleviate AP symptoms.

Accumulating evidence is available demonstrating that severe AP is associated with changes in the microcirculation, gut permeability/motility, bacterial translocation, and activation of the gut-associated lymphoid tissue. Preventing AP-associated intestinal barrier disruption or inflammation might therefore be a key target for effective therapy (22). A possible nutraceutical approach to prevent intestinal barrier disruption is by intervening with anti-inflammatory food components such as specific dietary fibers. Dietary fibers may be fermented by the gut microbiota to produce health-promoting short-chain fatty acids (SCFAs) and modify intestinal barrier function (23). Therefore, consumption of dietary fibers represents a promising strategy to modulate the progression of AP. We have recently demonstrated that low-methoxyl lemon pectin (LMP) attenuated inflammatory responses and improved intestinal barrier integrity in experimental AP (2). Another family of nutritional molecules with supporting effect on barrier function and anti-inflammatory effect that might be instrumental for management of AP is inulin-type fructans (ITFs) (23–27). ITFs are a family of dietary fibers belonging to beta(2→1) linear fructan-type carbohydrate subgroup and have an impact on gastrointestinal functions, which is largely related to their biochemical and physiological attributes. In the gut, ITFs are rapidly fermented to produce SCFAs that exert some of the local and systemic effects of ITFs. In addition, ITFs have demonstrated clear chemistry or degree of polymerization (DP)-dependent effects (28). Previous studies have demonstrated that DP or chain length of ITFs that affects the magnitude of receptor activation and/or their site of fermentation in the gut determines their prebiotic and immune modulatory effects on immune cells, gut barrier function, and microbiota composition (23–27, 29). ITFs have been implicated in a variety of inflammatory and immunological dysfunctions. However, their potential effects have not been studied in the management of AP.

In this study, we examined and compared the effects of long-chain ITF IV and the more readily fermentable short-chain ITF I on their protective effects during experimentally induced AP. In a caerulein-induced AP mice model, we studied after ITF treatment severity of pancreatitis, the frequencies of infiltrating neutrophils, macrophages, and dendritic cells in the pancreas and colon as well as production of the cytokines TNF-α, IL-1β, and IL-10. Additionally, we studied colon integrity by determining expression of gut–epithelial tight junction (TJ) modulatory proteins and barrier reinforcing immunomodulatory antimicrobial peptides (AMPs). Finally, we investigated the signaling pathways and kinases modulated by ITFs to gain insight in the mechanisms by which ITFs modulate AP.

## Materials and Methods

### Fibers and Structural Characterization

The applied ITF I (frutalose OFP, 2 < DP < 25) and ITF IV (FrutafitTEX, 10 < DP < 60) were extracted from chicory roots (Sensus B.V., Roosendaal, The Netherlands). Their specific chain length profiles (range and distribution) were characterized by high-performance anion exchange chromatography as previously described (23, 24).

### Animals

All animal-related experimental protocols were approved by the Institutional Animal Ethics Committee of Jiangnan University in compliance with the recommendations of national and international guidelines for the Care and Use of Laboratory Animals, and were performed in accordance with the guidelines therein. Eight-week-old female BALB/c mice, 20 ± 2 g (Su Pu Si Biotechnology, Suzhou, Jiangsu, China) reared on *ad-libitum* access to standard laboratory chow and water were used in this study. The animals were maintained at *Animal Housing Unit* of the University under controlled temperature (23–25°C), pathogen-free conditions, and at a 12:12 h light:dark cycle.

### Experimental Design and AP Induction

One-week time was allowed for the acclimatization of animals before starting the experiment(s). Mice were randomly divided into four groups (*n* = 4–6) according to different diets and fed for 72 h (Table 1).

**Table 1:** Different groups of animals with their corresponding diet used in this study.

Group	Diet
Control	Normal diet without AP
AP	Normal diet with AP
ITF IV + AP	5% ITF IV (in normal diet, w/w) with AP
ITF I + AP	5% ITF I (in normal diet, w/w) with AP

*AP, acute pancreatitis; ITF IV, inulin-type fructan IV (long-chain inulins); ITF I, inulin-type fructan I (short-chain inulins)*.

After 12 h of fasting, AP was induced in mice by eight repeated intraperitoneal (i.p.) injections of caerulein (50 µg/kg/h) (Sigma-Aldrich, St. Louis, MO, USA). The littermates in control group were injected (i.p.) with the same volume of normal saline and served as controls.

### Tissues Sampling

Mice were euthanized and sacrificed with a lethal dose of pentobarbitone sodium (100 mg/kg) 1 h after the last caerulein injection. For serum analysis, blood samples were centrifuged at 3,000 × *g* for 15 min, after which serum was collected and stored at –80°C. Tissues, including pancreas and colon, were excised, fixed in 4% paraformaldehyde or snap freeze in liquid nitrogen and stored at –80°C for later analysis.

### Serum Amylase and Lipase Measurements

Serum amylase activities were measured by a serum assay kit (Jian Cheng Bioengineering Institute, Nanjing, China) (30). Lipase activities were measured by enzyme-linked immunosorbent assay (ELISA) kits (R&D Systems, Minneapolis, MN, USA).

### Edema and Myeloperoxidase (MPO) Activity Measurement

A portion of freshly harvested pancreatic tissue was trimmed of fat and weighed. Pancreatic water contents were evaluated by the ratio of initial weight (wet weight) of the pancreas to its weight after drying at 80°C for 48 h (dry weight). MPO activity measurements were determined by an MPO assay kit (Jian Cheng Bioengineering Institute, Nanjing, China).

### Histological Evaluation

Fresh pancreatic and colonic tissues were fixed in 4% paraformaldehyde overnight, washed with ddH_2_O, and rehydrated with ethanol and embedded in paraffin. The Skiving machine Slicer PM2245 (Leica, Wetzlar, Germany) diced 5-µm sections were stained with hematoxylin and eosin (H&E). For pancreatic injury evaluation, a DM2000 light microscope (Leica, Wetzlar, Germany) was used at ×40 magnification. The examination was carried out based on both infiltrating inflammatory cells and other morphological changes in tissues which are considered to be markers of inflammation/tissue damage.

### ELISA Analysis

The levels of cytokines (TNF-α, IL-1β, and IL-10) in serum were measured by ELISA kits (R&D Systems, Minneapolis, MN, USA) following the standard procedure of the manufacturer. To measure the cytokines level of pancreatic and colonic tissues (TNF-α, IL-1β, and IL-10), the tissues were homogenized in a saline solution (1:19, w/v) using a Polytron homogenizer (Scientz-48, Ningbo, Zhejiang, China) at 55 Hz for 1 min. Samples were centrifuged at 4°C, 10,000 × *g* for 10 min. Finally, the supernatant was collected for ELISA analysis.

### RNA Isolation and Real-time Quantitative Polymerase Chain Reaction (RT-qPCR) Analysis

Transcription of mRNA of occludin, zonula occludens protein-1 (ZO-1), β-defensin-1 (DEFB1), and cathelicidin-related antimicrobial peptide (CRAMP) was analyzed by RT-qPCR. Total RNA was isolated from pancreas and colonic tissues using TRIzol (Life Technologies, MA, USA) and was subjected to reverse transcription using Prime-Script RT reagent kit (TaKaRa Bio, Japan) following the manufacturer’s instructions. SYBR^®^ Green RT-qPCR was performed using real-time PCR system (BIO RAD CFX Connect, CA, USA). The relative mRNA levels were normalized to mRNA levels of β-actin (housekeeping control) and calculations for fold change of each mRNA were made on comparative cycle threshold method (2^−ΔΔCt^). The primers used in this study are provided in Table 2.

**Table 2:** List of primers used for RT-qPCR.

Gene	Forward (5′–3′)	Reverse (5′–3′)
β-actin	GGCTGTATTCCCCTCCATCG	CCAGTTGGTAACAATGCCATGT
ZO-1	CTTCTCTTGCTGGCCCTAAAC	TGGCTTCACTTGAGGTTTCTG
Occludin	CACACTTGCTTGGGACAGAG	TAGCCATAGCCTCCATAGCC
DEFB1	CACATCCTCTCTGCACTCTGGAC	CCATCGCTCGTCCTTTATGCCATTC
CRAMP	GCTGTGGCGGTCACTATCAC	TGTCTAGGGACTGCTGGTTGA

*DEFB1, β-defensin-1; CRAMP, cathelicidin-related antimicrobial peptide; RT-qPCR, real-time quantitative polymerase chain reaction*.

### Western Blot Analysis

Pancreatic and colonic tissues were homogenized in ice-cold lysis buffer RIPA (containing cocktail protease inhibitors; Beyotime, Shanghai, China). Samples were centrifuged at 4°C, 10,000 × *g* for 10 min and equal amounts of protein (30 µg), as determined using standard bicinchoninic acid assay (BCA) method by BCA Protein Assay Kit (Beyotime, Shanghai, China). Samples were electrophoresed on blots 10% sodium dodecyl sulfate polyacrylamide gel electrophoresis gel and transferred to polyvinylidene difluoride membranes. Membranes were blocked with blocking buffer for 1.5 h at room temperature, washed with TBS-Tween 20 (TBST), and finally incubated overnight at 4°C with anti-extracellular signal-regulated kinase (ERK)/phospho-ERK, JNK/phospho-JNK, p38/phospho-p38, IRAK-4, phospho-p65, and GAPDH (housekeeping) antibodies. Incubation with fluorescently labeled secondary horseradish peroxidase (HRP)-conjugated secondary antibodies (1:3,000) was performed for 2 h at room temperature and immunoreactivity was analyzed by Western Lightening Plus enhanced chemiluminescence (PerkinElmer, MA, USA) according to the manufacturer’s instructions.

### Flow Cytometry Analysis

Freshly harvested pancreatic and colonic tissues were digested in 1.0 mg/mL collagenase-P (Boehringer, Mannheim, Germany) solution at 37°C for 15 min and filtered through 75 µm filters with hank’s solution (Beyotime, Shanghai, China). Single-cell suspensions were incubated for 30 min at 4°C in hank’s solution with the following mAbs: APC Rat Anti-Mouse CD11b, BV421 Rat Anti-Mouse F4/80, Alexa Fluor 700 Rat Anti-Mouse Ly-6G, PE Hamster Anti-Mouse CD11c, FITC Rat Anti-Mouse MHCII, and PerCP-Cy5.5 Rat Anti-Mouse CD8a (BD Pharmingen, CA, USA). Gating method of fluorescence-activated cell sorting was programmed as CD11b^+^ Ly-6G^+^ (for neutrophils), CD11b^+^ F4/80^+^ (for macrophages), CD11c^+^ MHCII^+^ (for conventional dendritic cells, cDCs), and CD8a^+^CD11c^+^ MHCII^+^ (for plasmacytoid dendritic cells, pDCs). Flow cytometer was performed on Attune NxT (Thermo Fisher Scientific, MA, USA). Data were analyzed using ACEA NovoExpress software (Novo Express International, Inc., South San Francisco, CA, USA).

### Statistical Analysis

Data are expressed as means ± SEM. The parametric distribution of the results was confirmed using Kolmogorov–Smirnov test. Statistical analysis was performed by one-way analysis of variance (ANOVA) followed by Tukey’s *post hoc* test using GraphPad Prism (version 5; GraphPad Software Inc., San Francisco, CA, USA). The values of *P* < 0.05 were considered to indicate a statistically significant difference.

## Results

### ITF IV Supplementation of the Diet Alleviates Severity of Caerulein-Induced AP

**Figure 1:**
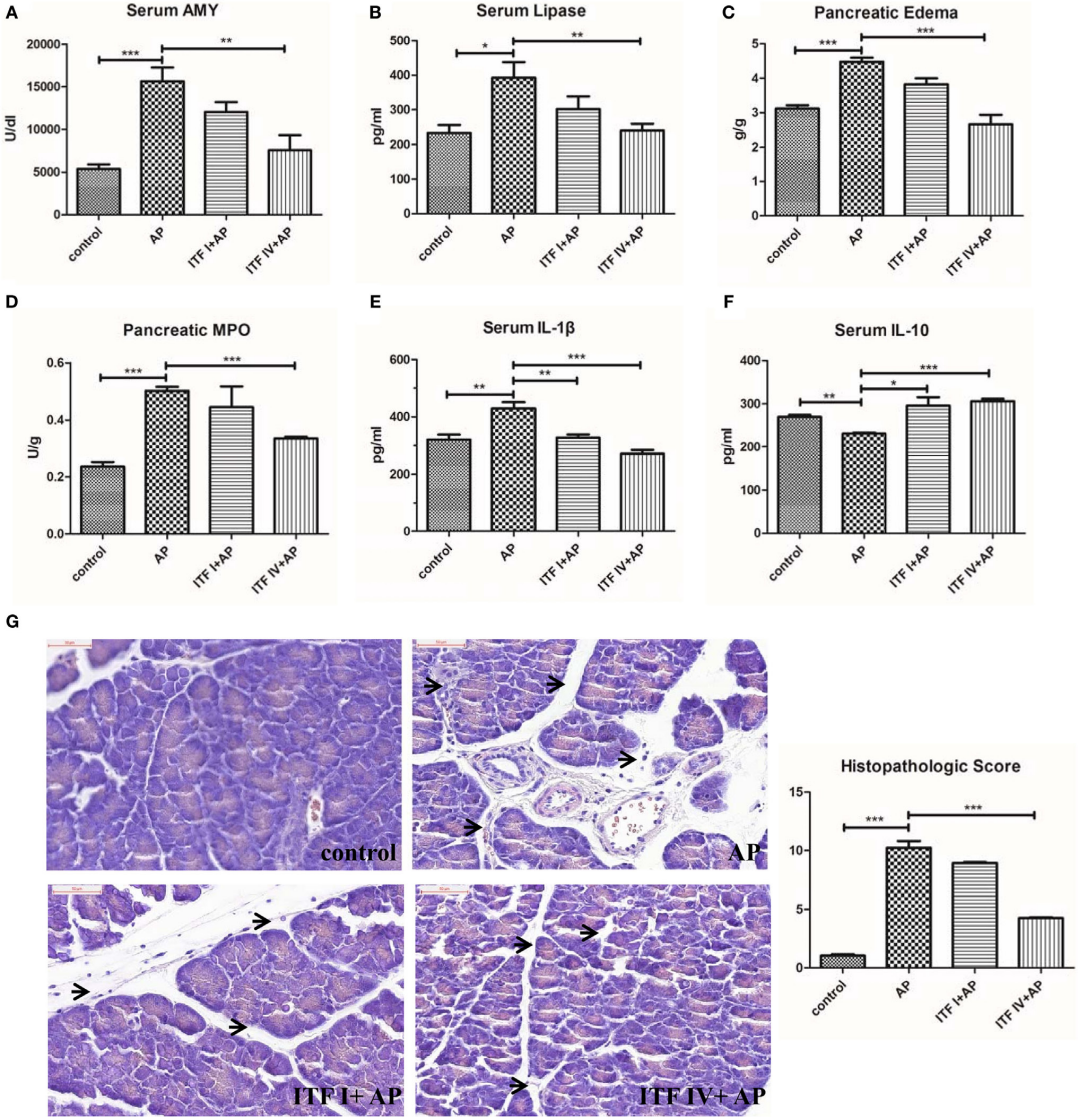
ITF IV attenuating the severity of AP. Mice were fed with 5% ITF I or ITF IV supplemented diets for 72 h before AP induction by caerulein. Serum amylase **(A)**, serum lipase **(B)**, pancreatic edema **(C)**, MPO activity **(D)**, serum IL-1β **(E)**, and IL-10 levels **(F)** were then determined as described in Section “Materials and Methods,” respectively. Representative photographs showed histomorphology of pancreatic tissues by H&E staining for the indicated groups (bar = 50 µm) and pancreatitis histopathologic score **(G)**. Data shown are means ± SEM. *P < 0.05, **P < 0.01, and ***P < 0.001. AP, acute pancreatitis; H&E, hematoxylin and eosin; IL, interleukin; ITF, inulin-type fructans; MPO, myeloperoxidase.

Modulatory effects of ITFs were examined in caerulein hyperstimulated mice by supplementing the diets with either ITF I or ITF IV for 3 days before AP induction. While dietary supplementation with 5% ITF I or ITF IV alone without AP induction did not show any effect on pancreatic markers (Figure S1 in Supplementary Material), protective effects of ITF supplementation on AP were observed and the effects were highly ITF chain length dependent. ITF IV had more attenuating effects than ITF I. ITF IV but not ITF I attenuated caerulein-induced increases in serum amylase, serum lipase, pancreatic MPO activities, and edema (Figures 1A–D). However, both ITF I and ITF IV modulated serum cytokines. Both types of ITFs reduced serum pro-inflammatory IL-1β levels and increased the regulatory cytokine IL-10 (Figures 1E,F). Histological examination of pancreatic sections confirmed an overall better attenuating AP effect of ITF IV as evidenced by generally improved cellular morphology, restored interlobular space expansion, reduced inflammatory infiltrates, and acini necrosis (Figure 1G). Taken together, these data indicate that ITF-IV-supplemented diets alleviate the severity of AP in mice, while ITF I diet has a more minor beneficial effect.

### ITF IV but Not ITF I Supplementation Strengthening Intestinal Barrier Function by Upregulating TJ Proteins and AMPs

**Figure 2:**
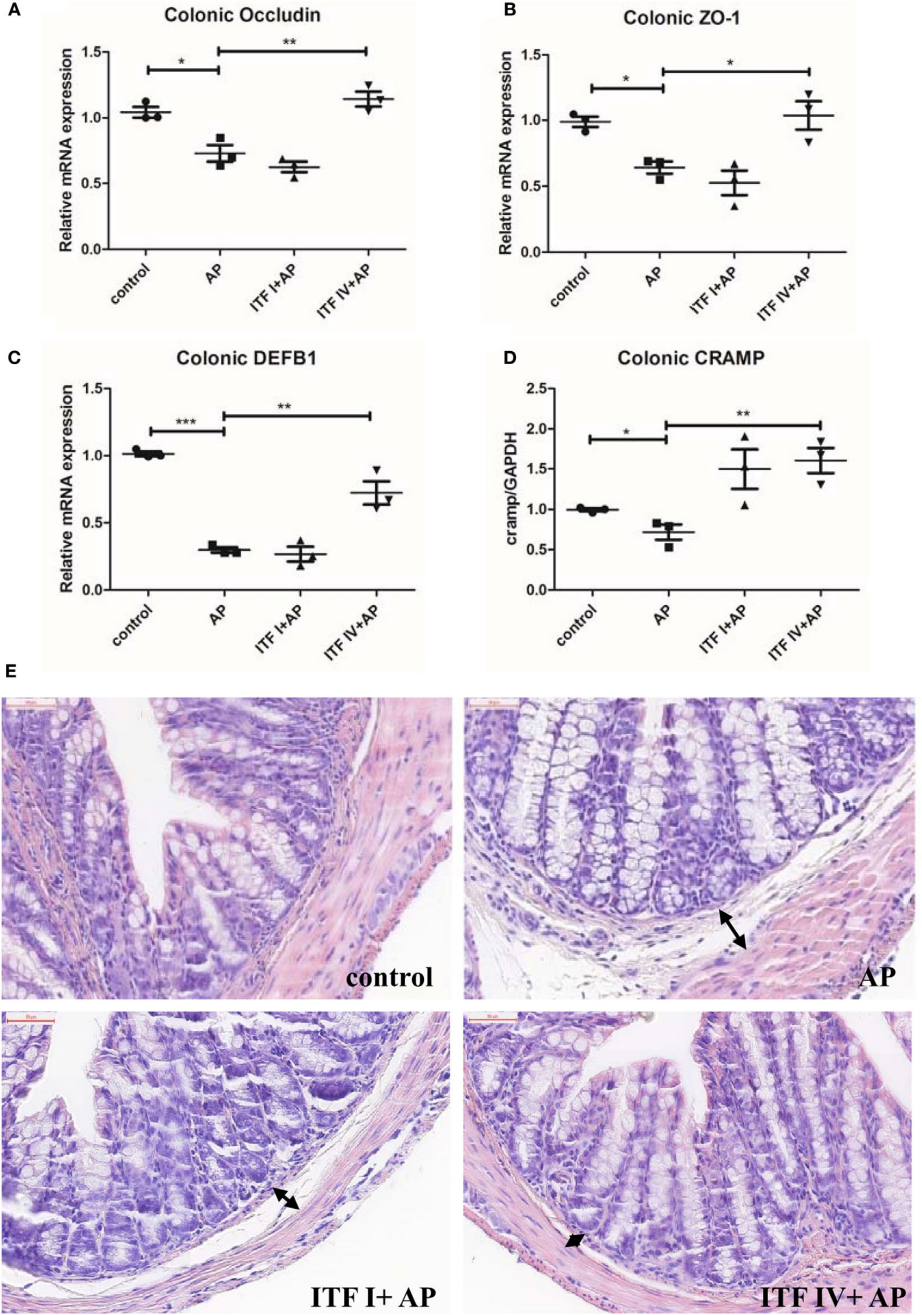
ITF IV upregulating TJ proteins and AMPs in the colon. Mice were fed with 5% ITF I or ITF IV supplemented diets for 72 h before AP induction by caerulein. The mRNA levels of TJ proteins occluding **(A)**, ZO-1 **(B)**, DEFB1 **(C)**, and CRAMP **(D)** in the colon were then determined by RT-qPCR analyses. Representative photographs showed histomorphology of colonic tissues (50 µm) by H&E staining **(E)**. Data shown are means ± SEM. *P < 0.05, **P < 0.01, and ***P < 0.001. AMPs, antimicrobial peptides; AP, acute pancreatitis; CRAMP, cathelicidin-related antimicrobial peptide; DEFB1, β-defensin-1; H&E, hematoxylin and eosin; ITF, inulin-type fructans; RT-qPCR, real-time quantitative polymerase chain reaction; SEM, standard error of mean; TJ, tight junction; ZO-1, zonula occludens-1.

Compromised intestinal barrier integrity and intestinal injury, marked by dysregulated expression of TJ modulatory proteins and barrier reinforcing AMPs, develop as AP progresses (22, 31). Therefore, maintaining intestinal barrier function might be key in the ITF-induced attenuating effects on AP. To determine such an effect of ITFs, we measured in ITF-treated animals, the mRNA expression of major structural proteins such as TJ, occludin and ZO-1, as well as AMPs DEFB1 and CRAMP. All these molecules are involved in the physical and chemical barriers of the mucosa (32, 33). The RT-qPCR analysis showed that ITF IV but not ITF I supplementation prevented caerulein-induced downregulation of occludin and ZO-1 as well as the downregulation of DEFB1 and CRAMP (Figures 2A–D). Furthermore, histological examination of colon sections revealed that intestinal mucosal epithelia widened and villous apical epithelium peeled off in mice with AP as compared with control mice. This colonic damage was less severe in mice fed on ITF IV supplemented diet (Figure 2E). Together, these findings suggest that ITF IV but not ITF I supplementation prevents AP-associated gut integrity damage and injury.

### ITF IV Supplementation Reducing Innate Immune Cell Infiltration into the Pancreas and Pancreatic Cytokine Production during AP

**Figure 3:**
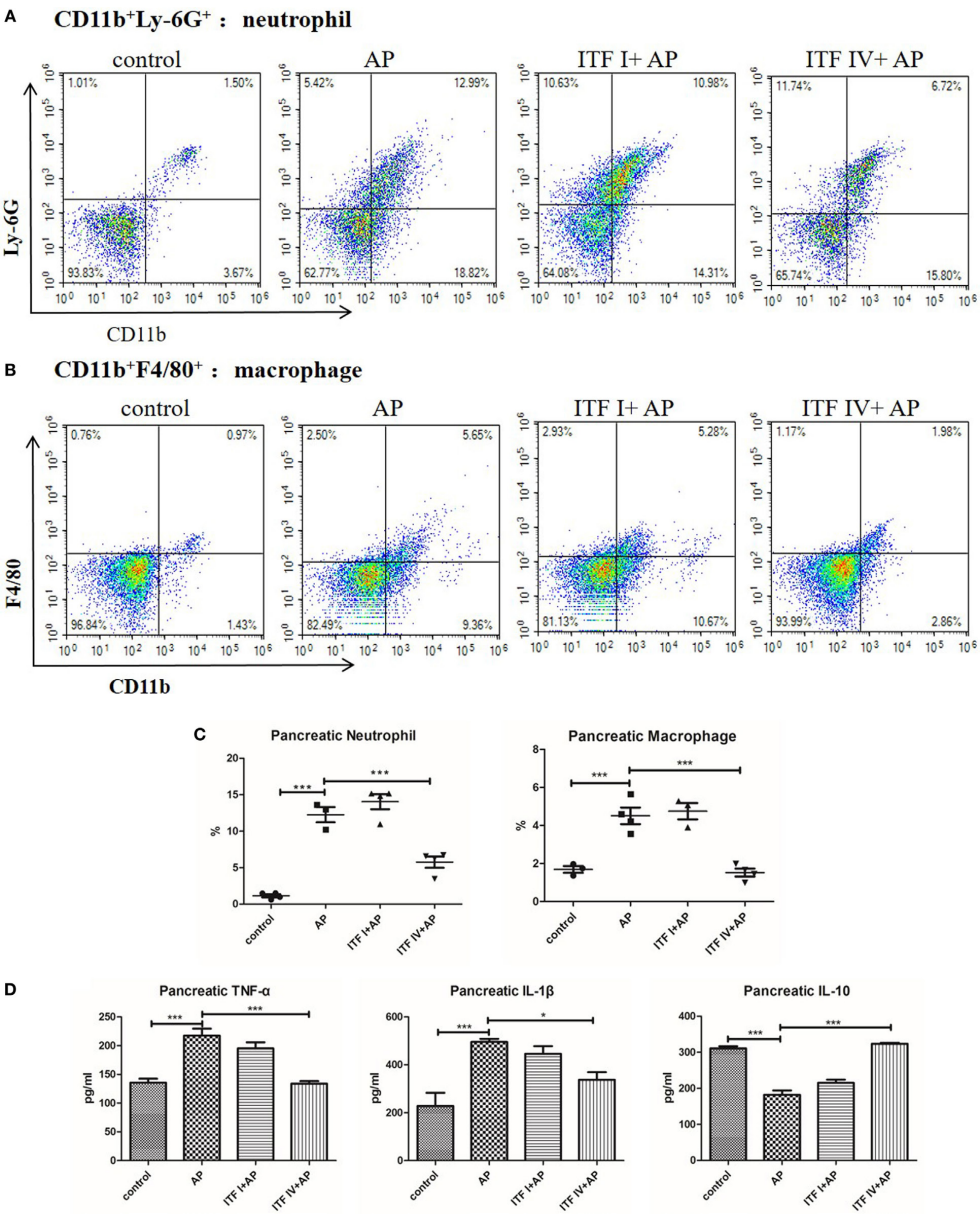
Effects of ITF IV on AP-mediated neutrophil and macrophage infiltration and cytokines production in the pancreas. Mice were fed with 5% ITF I or ITF IV supplemented diets for 72 h before AP induction by caerulein. Neutrophil and macrophage infiltration and cytokine production in the pancreas were determined as described in Section “Materials and Methods,” respectively. Representative graphs showed dot plots of Ly-6G^+^CD11b^+^ neutrophils **(A)** and F4/80^+^CD11b^+^ macrophages **(B)** in the pancreas and quantitative analysis of neutrophils and macrophages infiltration **(C)**. Quantitative analyses of TNF-α, IL-1β, and IL-10 levels in the pancreas were performed by ELISA **(D)**. Data shown are means ± SEM. *P < 0.05 and ***P < 0.001. AP, acute pancreatitis; ELISA, enzyme-linked immunosorbent assay; IL, interleukin; ITF, inulin-type fructans; TNF-α, tumor necrosis factor-α.

Both infiltration of inflammatory immune cells and an imbalance between pro-inflammatory and anti-inflammatory cytokines are key immunological pathophysiological events in AP development (34–36). For this reason, we determined the frequencies of neutrophils (CD11b^+^Ly-6G^+^), macrophages (CD11b^+^F4/80^+^), cDCs (CD11c^+^MHCII^+^), and pDCs (CD8a^+^ CD11c^+^MHCII^+^) that infiltrated into the pancreas of AP mice fed with ITFs. ITF IV supplementation profoundly inhibited the infiltration of neutrophils and macrophages (Figure S2 in Supplementary Material). The percentage of CD8a^+^ DCs or pDCs but not cDCs was lowered with AP but normal in mice fed with ITF IV. ITF I did not have such an effect (Figure S2 in Supplementary Material). The percentages of neutrophil and macrophage in the pancreas robustly increased with AP induction and were significantly less in mice fed with ITF IV (Figures 3A–C) but this was still increased in mice fed with ITF I. Additionally, pancreatic cytokine production remained enhanced in ITFI but not in animals fed with ITF IV (Figure 3D). Collectively, these data demonstrate the immune attenuating effects of ITF IV.

### Both ITF IV and ITF I Supplementation Modulating AP-Induced Innate Immune Cell Infiltration in the Gut and Cytokine Production

**Figure 4:**
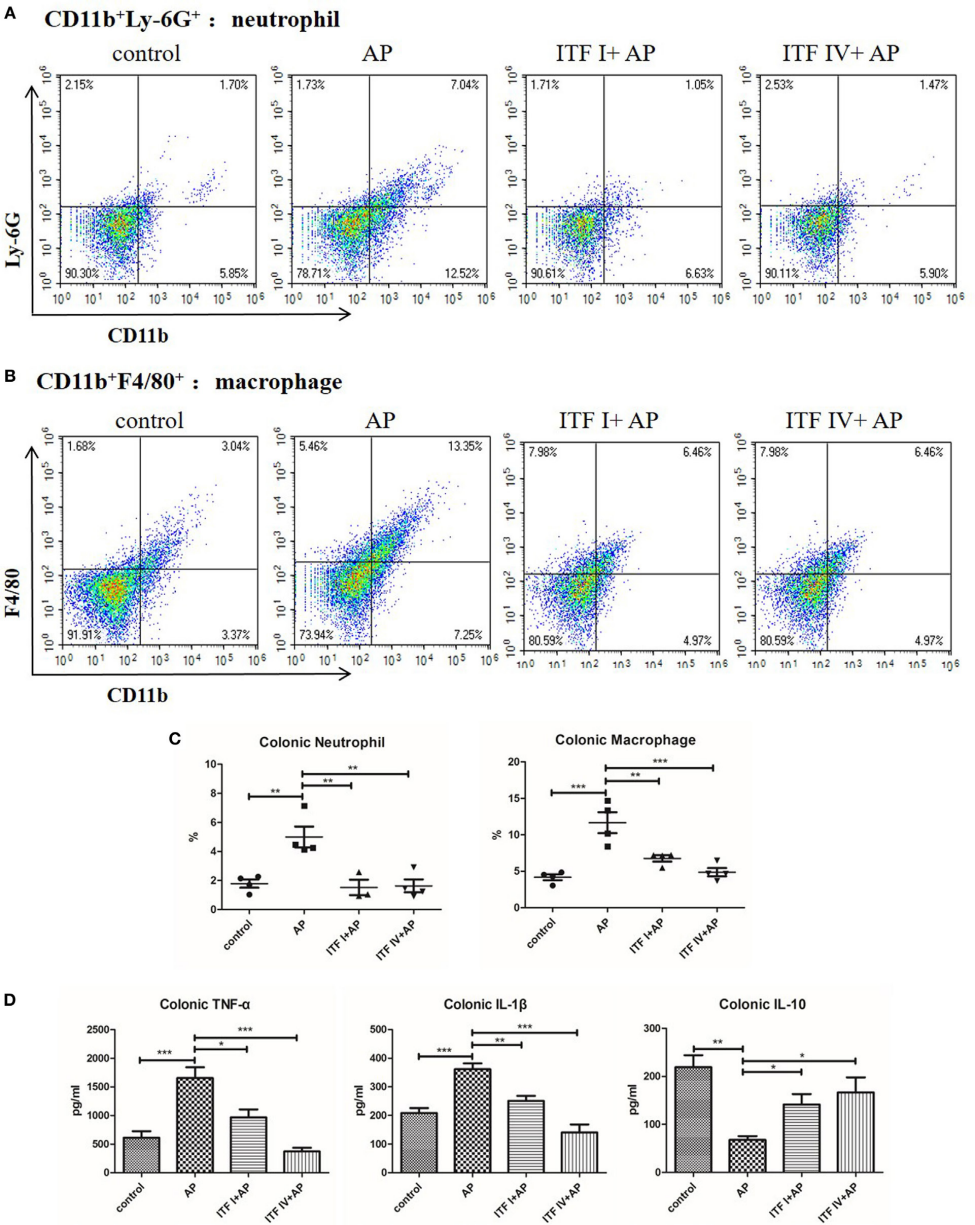
Effects of ITFs on neutrophil and macrophage infiltration and cytokine production in the colon. Mice were fed with 5% ITF I or ITF IV supplemented diets for 72 h before AP induction by caerulein. Neutrophil and macrophage infiltration and cytokine production in the colon were determined as described in Section “Materials and Methods,” respectively. Representative graphs showed dot plots of Ly-6G^+^CD11b^+^ neutrophils **(A)** and F4/80^+^CD11b^+^ macrophages **(B)** in the pancreas and quantitative analysis of neutrophils and macrophages infiltration in the colon **(C)**. Quantitative analyses of TNF-α, IL-1β, and IL-10 levels in the colon were performed by ELISA **(D)**. Data shown are means ± SEM. *P < 0.05, **P < 0.01, and ***P < 0.001. AP, acute pancreatitis; ELISA, enzyme-linked immunosorbent assay; IL, interleukin; ITF, inulin-type fructans; TNF-α, tumor necrosis factor-α.

Immune compromised gut environment as a result of disrupted barrier function and intestinal injury is associated with major inflammatory cell infiltration and imbalanced inflammatory cytokine production. Therefore, we also investigated infiltration of innate immune cells in the gut during AP. AP induction resulted in robust increases in neutrophils and macrophages in the colon. Both ITF I and ITF IV supplementation reduced the number of infiltrating neutrophils and macrophages (Figures 4A–C). The percentage of CD8a^+^ DCs or pDCs in the colon lowered with AP, but was prevented by ITF IV and not by ITF I (Figure S3 in Supplementary Material). Furthermore, consistent with reduced inflammatory cell infiltration in the gut, both ITFs prevented AP-induced increases in pro-inflammatory TNF-α and IL-1β levels, and decreases in IL-10 level in the colon as compared with untreated AP control (Figure 4D). Taken together, these data suggest positive immunomodulatory effects of both ITFs on AP-associated gut inflammation.

### ITF IV but Not ITF I Supplementation Modulating Pancreatic IRAK-4/JNK/NF-κB Activation in AP

**Figure 5:**
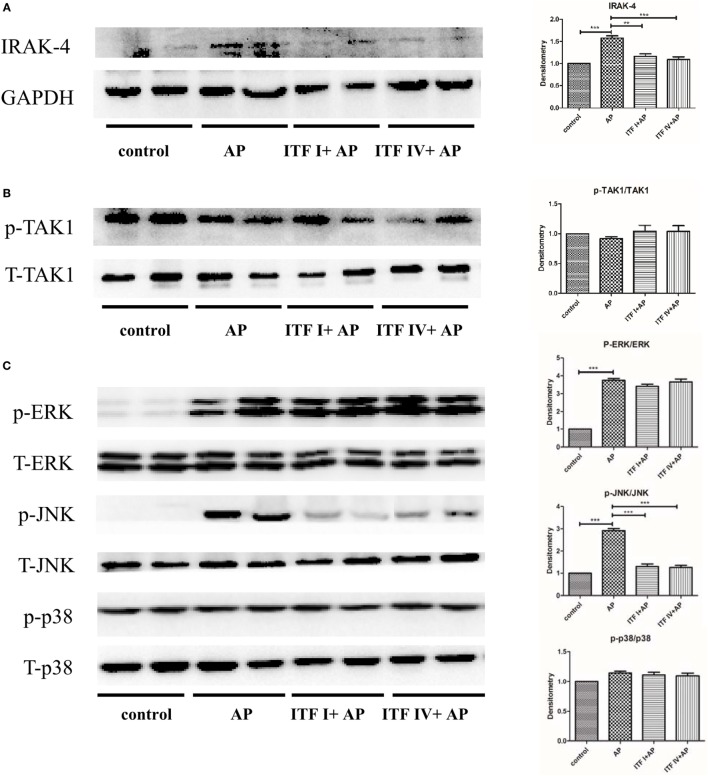

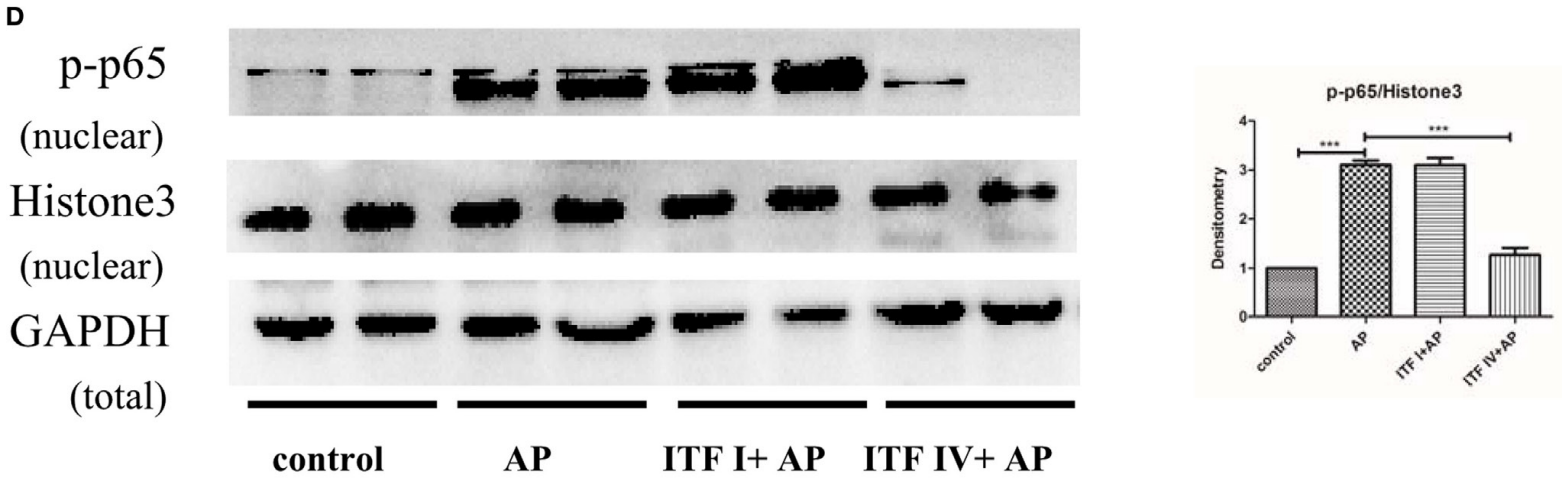
Effects of ITFs on AP-mediated activation of MAPK and NF-κB pathways in the pancreas. Mice were fed with 5% ITF I or ITF IV supplemented diets for 72 h before AP induction by caerulein. The activation/expression of pancreatic IRAK4 **(A)**, p-TAK1 **(B)**, p-ERK, p-JNK, p-p38 **(C)**, and NF-κB p-p65 **(D)** were examined by Western blot. Data shown are means ± SEM. **P < 0.01 and ***P < 0.001. AP, acute pancreatitis; ERK, extracellular signal-regulated kinase; IRAK, IL-1 receptor associated kinase; ITF, inulin-type fructans; JNK, c-Jun N-terminal kinase; MAPKs, mitogen-activated protein kinases; NF-κB, nuclear factor kappa-light-chain-enhancer of activated B cells; TAK, TGF-β activated kinase.

Lastly, we investigated the signaling molecular pathways underlying differential effects by ITF I and ITF IV supplementation. Activation of NF-κB and its upstream signaling kinases (IRAK, TAK1, and MAPKs) were determined by Western blot. It was found that ITF IV significantly modulated AP-induced IRAK-4, JNK, and NF-κB activation, while p-TAK, p-ERK, and p-p38 remained unaffected by ITF (Figures 5A–D). In comparison, ITF I attenuated IRAK-4 and p-JNK activation associated with AP, but did not affect NF-κB nuclear translocation and activation (Figures 5A–D).

## Discussion

**Figure 6:**
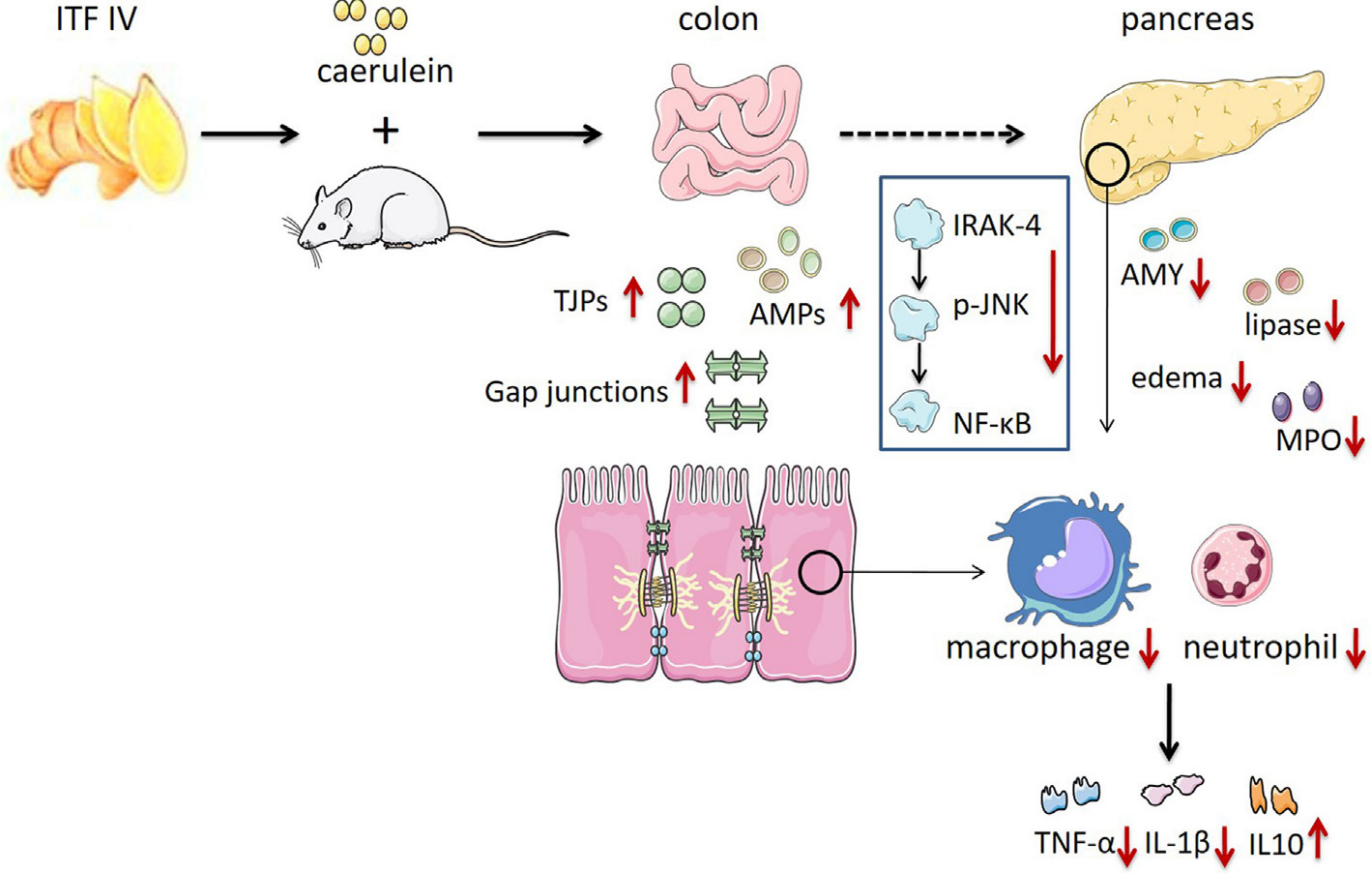
ITF IV protection against AP by reducing pancreatic damage and AP-associated intestinal injury preventing its progression into a systemic inflammatory response. The role of ITF IV on AP in preventing tissues damage and the subsequent inflammatory responses was illustrated. AP, acute pancreatitis; ITF, inulin-type fructans.

The current study examines and compares effects of different types of ITFs in experimental AP and associated intestinal immune and barrier dysregulation. The effects were ITF type dependent and more pronounced with ITF IV than with ITF I. We observed that dietary long-chain inulin (ITF IV) supplementation mitigated the severity of AP: it suppressed characteristic inflammatory cell infiltration, modulated inflammatory cytokine production in the pancreatic–gut region, and prevented intestinal barrier function integrity. Its attenuating effect was associated with suppression of AP-induced IRAK-4/p-JNK/NF-κB pathway activation. ITF I only demonstrated significant modulatory effects in colonic immune responses and serum inflammatory markers, suggesting that short-chain inulin effects are limited to the gut (Figure 6).

Inulin-type fructans have been tested in a variety of inflammatory and immunological disease models and it has been shown that administration of ITF can exert immunomodulatory actions and alleviate acute inflammation (23, 28, 37). To the best of our knowledge, this is the first study in which efficacy of ITF is shown in AP. It has been earlier suggested, however, that dietary fibers are inversely related with pancreatic enzyme activity and can inhibit pancreatic digestive enzyme activities *in vivo* (38). As premature activation of pancreatic zymogens is a triggering event of human AP and inhibited by ITF, this study supports the potential application of ITF in preventing AP.

A particular intriguing observation of this study was that long-chain inulins have a better local pancreatic and gut protective effect in AP than short-chain inulins. Previously, differential prebiotic and immunomodulatory effects have been demonstrated by ITFs of varying chain length. Prolonged fermentation of long-chain inulin (DP > 10) compared with short-chain inulin (DP < 10) ensures more endurable and profound prebiotic and immunomodulatory effects, locally in the colon and systemically in remote organs. Another ITF chain length-dependent mechanism which might explain the difference is the magnitude by which ITFs can activate toll-like receptor 2, which is higher with long-chain than with short-chain ITFs (24). In addition, the preferential sites of fermentation for these two ITFs are different: colon for long-chain and small intestine for short-chain inulins. This may imply a prolonged and different action of ITF IV as compared with ITF I and might explain the observation that ITF IV but not ITF I increased the colonic expression of the TJ proteins ZO-1 and occluding as well as AMP DEFB1 and CRAMP. Differential regulation of TJ proteins has been observed after dietary interventions earlier (39).

Innate immune activations and pancreatic acinar cell inflammatory signaling are characteristic for AP (7). Moreover, AP induction by caerulein hyperstimulation caused neutrophil and macrophage infiltration. This infiltration is prevented by dietary ITF IV supplementation. Increased pancreatic Ly6C^hi^ monocytes/macrophages have been found to be positively correlated with the severity of AP and are dependent on TNF-α production by these cells (11). IL-10 production by M2 macrophages has been shown to impair neutrophil recruitment in inflammatory and infectious conditions (40, 41). Along with reduced infiltration of neutrophils and macrophages in ITF IV-fed mice with AP, the production of TNF-α and IL-1β was found to be suppressed and IL-10 levels increased in pancreatic and colonic tissues. While modulatory effects of ITF IV were observed on pancreatic, colonic, and systemic cytokine levels, those of ITF I was only significant in colonic and systemic cytokines. Balanced pro-inflammatory and anti-inflammatory cytokine production prevented an augmented inflammatory response from a positive inflammatory feedback circuit (42).

Pancreatic acinar inflammation with activation of MAPKs and of the transcription factor NF-κB is a key pathological event during the development of AP (43–46). AP induction or supraphysiological concentrations of caerulein are associated with MAPK activation in pancreatic acini (47). Overexpression of p65 protein trans-activated NF-κB aggravates acute pancreatic inflammatory responses and MAPKs regulate NF-κB activation by mediating phosphorylation of its inhibitory-κB IκB protein, to allow NF-κB translocation to the nucleus followed by upregulation of inflammatory genes (19, 47, 48). Here, we found that MAPKs JNK but not ERK1/2 or p38 activation was inhibited by ITF IV, concomitant with an attenuated NF-κB p65 nuclear translocation and activation. It might be speculated that different upstream MAPKs are regulated by dietary fibers. Our data suggest that ITF IV interferes with the development of AP, by reducing pancreatic activation of IRAK-4-JNK-NF-κB p65 signaling pathway and inhibiting the release of inflammatory mediators. It has been demonstrated that IRAK-4 has been demonstrated essential in many innate immune responses and can activate JNK pathway and downstream NF-κB (20, 21, 49). NF-κB translocation in acinar cells increases the severity of pancreatitis in mice, and leads to transcription of cytokines and other inflammatory mediators (19). Our study shows that ITF IV downregulated IRAK-4/p-JNK/NF-κB p65 in the pancreas during AP.

As AP progresses, the inflammatory cytokines produced in the pancreas, including TNF-α and IL-1β, reach the gut by the microcirculation. These cytokines will recruit more leukocytes and induce more inflammatory mediators that ultimately will contribute to intestinal barrier dysfunction and mucosal injury (22, 50, 51). Gut barrier dysfunction and worsened gut permeability in AP are accompanied by reduced expression of TJ reinforcing proteins and AMPs. Lack of physical and chemical barriers of the gut makes the host susceptible to bacterial translocation that contributes to a second golf of inflammatory event in the pancreas, causing excessive inflammation and development of multi-organ dysfunctions. As shown here, dietary ITF IV intervention, by limiting pancreatic–gut inflammation, restoring gut barrier function and integrity to prevent secondary excessive inflammatory responses, represents a promising approach to prevent the progress of AP. Although we have shown the modulatory effects of ITF IV on pancreatic–intestinal immunity and gut barrier function, the detailed mechanisms remain to be fully understood. Future investigation could identify specific receptor involvement and/or examine how their fermentation product SCFAs are regulated to exert protective effects during the condition. Moreover, the translational importance of these safe, value-added prebiotics for clinical AP merits further exploration.

Collectively, our data suggest that dietary ITF IV but not ITF I intake prevents the development of AP by three-graded actions: first, by preventing pancreatic inflammation and damage; second, by preventing AP-associated intestinal barrier dysfunction and intestinal inflammation; and last, by precluding progression of the gut and pancreatic inflammation into a systemic inflammatory response by the two aforementioned mechanisms. The current study provides evidence that nutritional application of long-chain inulins in clinical AP might be efficacious.

## Ethics Statement

All animal-related experimental protocols were approved by the Institutional Animal Ethics Committee of Jiangnan University in compliance with the recommendations of national and international guidelines for the Care and Use of Laboratory Animals, and were performed in accordance with the guidelines therein.

## Author Contributions

YH, CW, JL, and HL performed experiments and analyzed data. P-LL, ZS, HZ, and PDV provided intellectual inputs, contributed to the data acquisition, and critically reviewed the manuscript. JS and P-LL designed and interpreted experiments. JS, P-LL, YH, and PDV wrote the paper.

## Conflict of Interest Statement

The authors declare that the research was conducted in the absence of any commercial or financial relationships that could be construed as a potential conflict of interest.

## Supplementary Material

Figure S1 Effects of dietary ITF supplementation on markers of pancreatic damage. Mice were fed with 5% ITF I or 5% ITF IV supplemented diets for 72 h. Serum amylase **(A)**, pancreatic MPO activity **(B)**, and pancreatic edema **(C)** were then determined as described in Section “Materials and Methods,” respectively. Data shown are means ± SEM. ITF, inulin-type fructans; MPO, myeloperoxidase.

Figure S2 Effects of ITF IV on CD8a^+^ DCs in the pancreas during AP: representative flow cytometry dot plots of CD11c^+^MHCII^+^ DCs **(A)**, histogram **(B)**, and percentage **(C)** of CD8a^+^ DCs on DC subsets in the pancreas were shown. Data shown are means ± SEM from four independent experiments with three to four pooled mice per group in each experiment. ***P* < 0.01. AP, acute pancreatitis; DCs, dendritic cells; ITF, inulin-type fructans.

Figure S3 Effects of ITF IV on CD8a^+^ DCs in the colon during AP: representative flow cytometer dot plots of CD11c^+^MHCII^+^ DCs **(A)**, histogram **(B)**, and percentage **(C)** of CD8a^+^ DCs on DC subsets in the colon were shown. Data shown are means ± SEM from four independent experiments with three to four pooled mice per group in each experiment. **P* < 0.05 and ***P* < 0.01. AP, acute pancreatitis; DCs, dendritic cells; ITF, inulin-type fructans.

## Abbreviations

AP, acute pancreatitis; AMPs, antimicrobial peptides; ANOVA, analysis of variance; CRAMP, cathelicidin-related antimicrobial peptide; DEFB1, β-defensin-1; DP, degree of polymerization; ERK, extracellular signal-regulated kinase; H&E, hematoxylin and eosin; IL, interleukin; IRAK, IL-1 receptor associated kinase; ITF, inulin-type fructan; JNK, c-Jun N-terminal kinase; LMP, low-methoxyl pectins; MAPKs, mitogen-activated protein kinases; MPO, myeloperoxidase; NF-κB, nuclear factor kappa-light-chain-enhancer of activated B cells; RT-qPCR, real-time quantitative polymerase chain reaction; SCFAs, short-chain fatty acids; TAK, TGF-β activated kinase; TGF-β, transforming growth factor-β; TJ, tight junction; TNF-α, tumor necrosis factor-α; ZO-1, zonula occludens-1.
