# Corrigendum: Inulin-Type Fructans Modulates Pancreatic-Gut Innate Immune Responses and Gut Barrier Integrity during Experimental Acute Pancreatitis in a Chain Length-Dependent Manner

**DOI:** 10.3389/fimmu.2018.00812

**Published:** 2018-04-18

**Authors:** Yue He, Chengfei Wu, Jiahong Li, Hongli Li, Zhenghua Sun, Hao Zhang, Paul de Vos, Li-Long Pan, Jia Sun

**Affiliations:** ^1^State Key Laboratory of Food Science and Technology, Jiangnan University, Wuxi, China; ^2^School of Food Science and Technology, Jiangnan University, Wuxi, China; ^3^Division of Medical Biology, Department of Pathology and Medical Biology, University of Groningen, University Medical Center Groningen, Groningen, Netherlands; ^4^School of Medicine, Jiangnan University, Wuxi, China

**Keywords:** dietary fibers, inflammation, pancreatic-intestinal immunity, signaling kinases, tight junction proteins, antimicrobial peptides

**Incorrect Affiliation**

In the published article, there was an error in affiliation [4,5]. Instead of “^4^Department of Pharmacology, School of Pharmacy, Fudan University, Shanghai, China, ^5^School of Medicine, Jiangnan University, Wuxi, China”, it should be “^4^School of Medicine, Jiangnan University, Wuxi, China”. The authors apologize for this error and state that this does not change the scientific conclusions of the article in any way.

The original article has been updated.

## Conflict of Interest Statement

The authors declare that the research was conducted in the absence of any commercial or financial relationships that could be construed as a potential conflict of interest.

