# Supplementary figures and images for: Inulin-Type Fructans Modulates Pancreatic–Gut Innate Immune Responses and Gut Barrier Integrity during Experimental Acute Pancreatitis in a Chain Length-Dependent Manner
Source: Front Immunol. 2017 Sep 26;8:1209. doi: 10.3389/fimmu.2017.01209 (PMC5622924; doi:10.3389/fimmu.2017.01209)

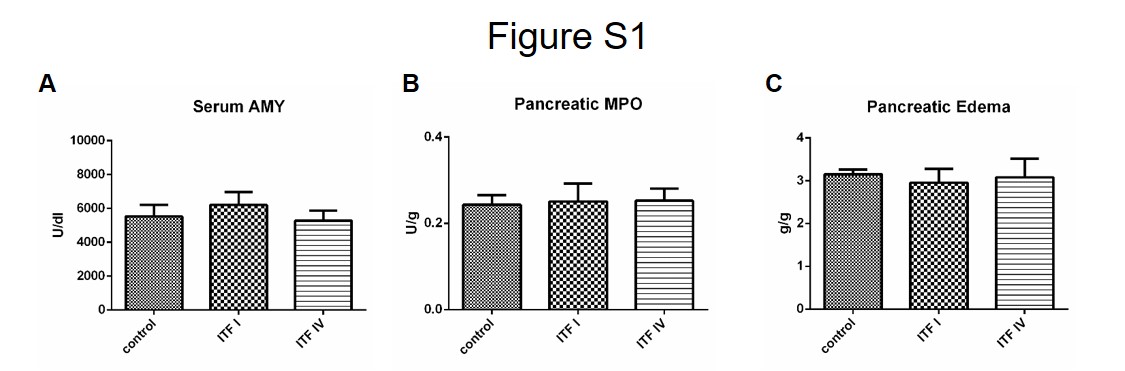

Supplement: Figure S1 — Effects of dietary ITF supplementation on markers of pancreatic damage. Mice were fed with 5% ITF I or 5% ITF IV supplemented diets for 72 h. Serum amylase (A), pancreatic MPO activity (B), and pancreatic edema (C) were then determined as described in Section “Materials and Methods,” respectively. Data shown are means ± SEM. ITF, inulin-type fructans; MPO, myeloperoxidase. [file image_1.jpeg]

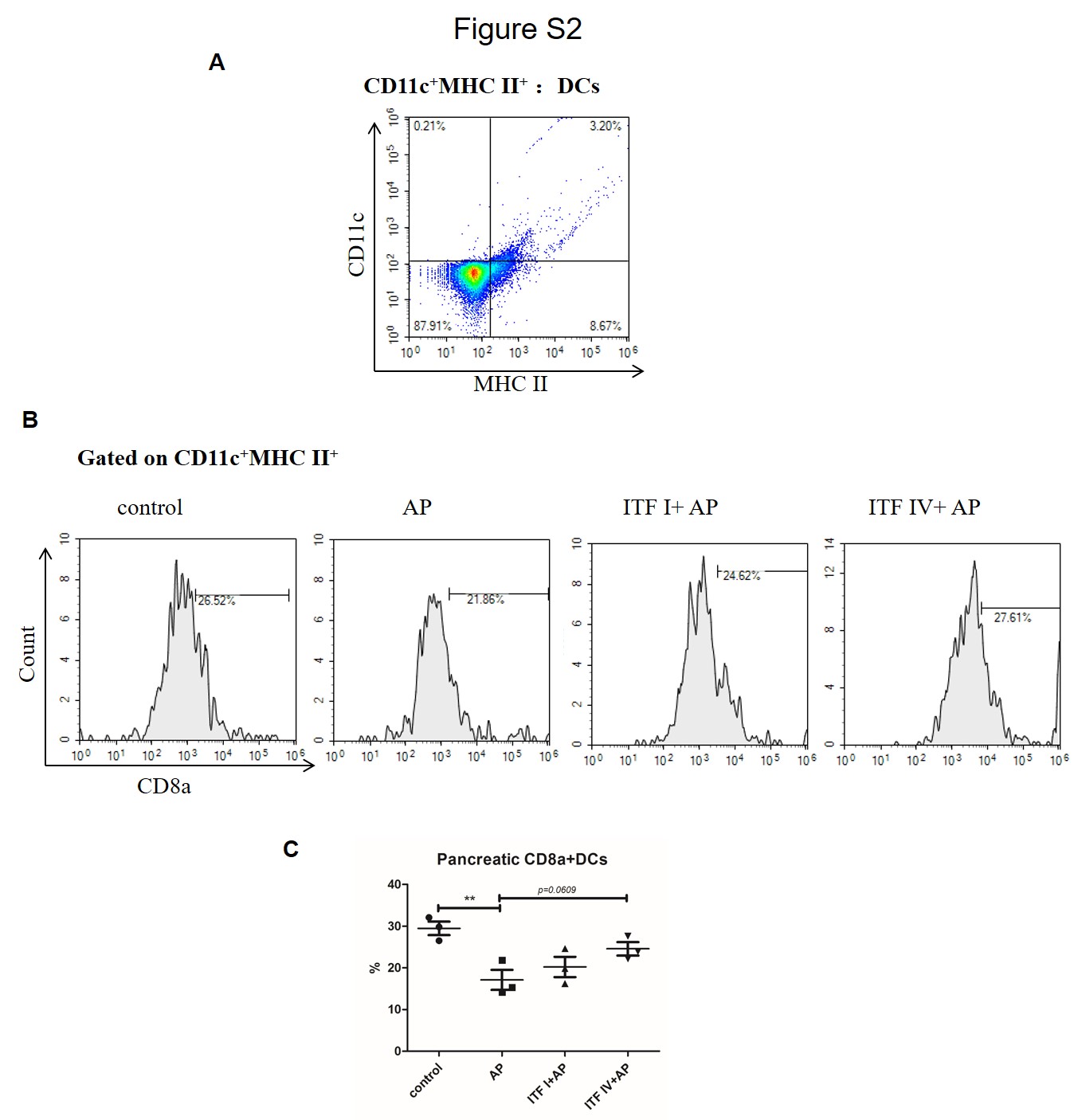

Supplement: Figure S2 — Effects of ITF IV on CD8a+ DCs in the pancreas during AP: representative flow cytometry dot plots of CD11c+MHCII+ DCs (A), histogram (B), and percentage (C) of CD8a+ DCs on DC subsets in the pancreas were shown. Data shown are means ± SEM from four independent experiments with three to four pooled mice per group in each experiment. **P < 0.01. AP, acute pancreatitis; DCs, dendritic cells; ITF, inulin-type fructans. [file image_2.jpeg]

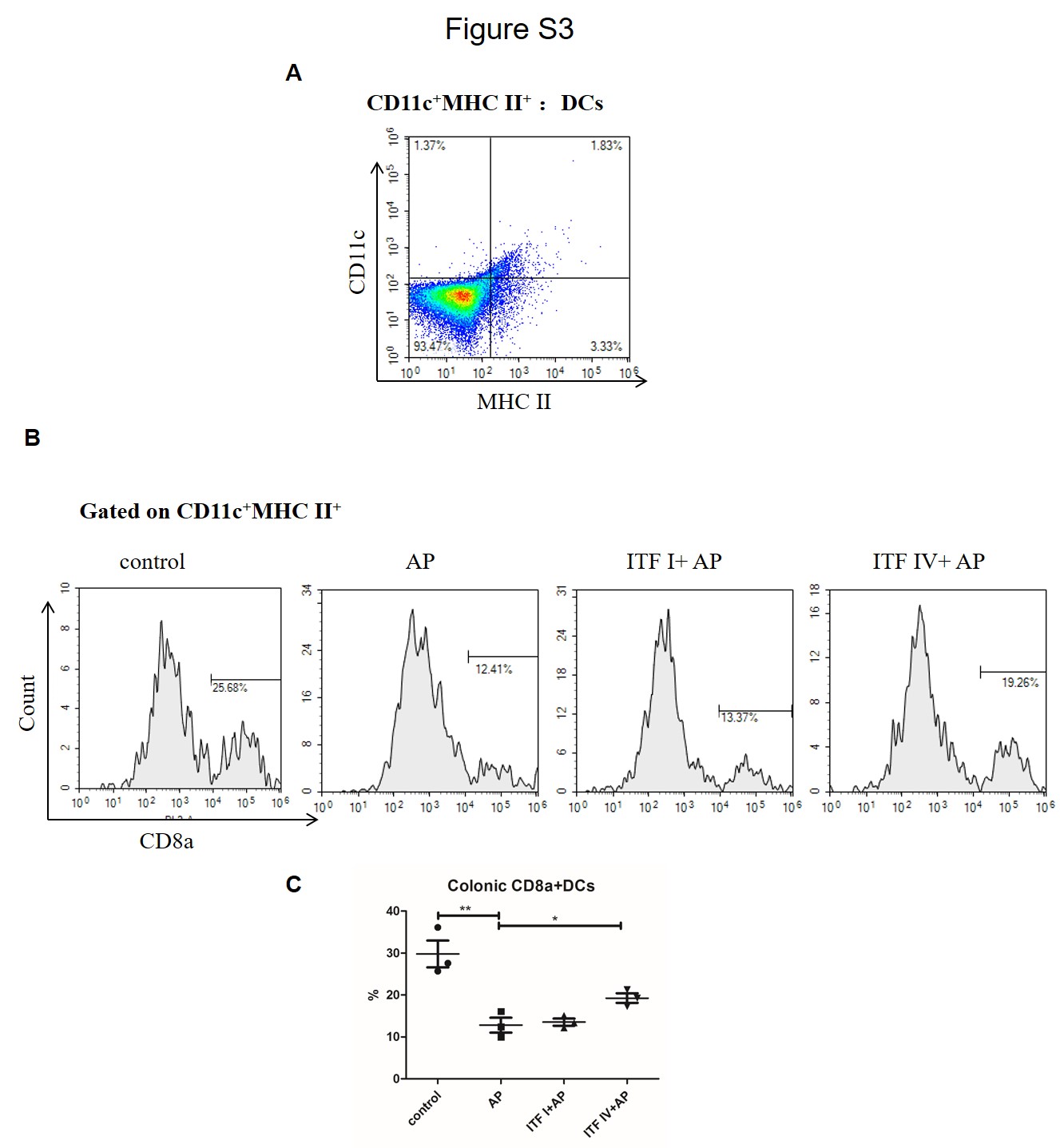

Supplement: Figure S3 — Effects of ITF IV on CD8a+ DCs in the colon during AP: representative flow cytometer dot plots of CD11c+MHCII+ DCs (A), histogram (B), and percentage (C) of CD8a+ DCs on DC subsets in the colon were shown. Data shown are means ± SEM from four independent experiments with three to four pooled mice per group in each experiment. *P < 0.05 and **P < 0.01. AP, acute pancreatitis; DCs, dendritic cells; ITF, inulin-type fructans. [file image_3.jpeg]
